# Small‐scale genetic structure of coral populations in Palau based on whole mitochondrial genomes: Implications for future coral resilience

**DOI:** 10.1111/eva.13509

**Published:** 2023-01-05

**Authors:** Stephen R. Palumbi, Nia S. Walker, Erik Hanson, Katrina Armstrong, Marilla Lippert, Brendan Cornwell, Victor Nestor, Yimnang Golbuu

**Affiliations:** ^1^ Department of Biology and Oceans Department Hopkins Marine Station of Stanford University Pacific Grove California USA; ^2^ Palau International Coral Reef Center Koror Palau; ^3^ Hawaii Institute of Marine Biology, University of Hawaii Honolulu Hawaii USA

**Keywords:** assisted gene flow, coral, dispersal, mitochondrial genomes, Palau, population genetics – empirical

## Abstract

The ability of local populations to adapt to future climate conditions is facilitated by a balance between short range dispersal allowing local buildup of adaptively beneficial alleles, and longer dispersal moving these alleles throughout the species range. Reef building corals have relatively low dispersal larvae, but most population genetic studies show differentiation only over 100s of km. Here, we report full mitochondrial genome sequences from 284 tabletop corals (*Acropora hyacinthus*) from 39 patch reefs in Palau, and show two signals of genetic structure across reef scales from 1 to 55 km. First, divergent mitochondrial DNA haplotypes exist in different proportions from reef to reef, causing Phi_ST_ values of 0.02 (*p* = 0.02). Second, closely related sequences of mitochondrial Haplogroups are more likely to be co‐located on the same reefs than expected by chance alone. We also compared these sequences to prior data on 155 colonies from American Samoa. In these comparisons, many Haplogroups in Palau were disproportionately represented or absent in American Samoa, and inter‐regional Phi_ST_ = 0.259. However, we saw three instances of identical mitochondrial genomes between locations. Together, these data sets suggest two features of coral dispersal revealed by occurrence patterns in highly similar mitochondrial genomes. First, the Palau‐American Samoa data suggest that long distance dispersal in corals is rare, as expected, but that it is common enough to deliver identical mitochondrial genomes across the Pacific. Second, higher than expected co‐occurrence of Haplogroups on the same Palau reefs suggests greater retention of coral larvae on local reefs than predicted by many current oceanographic models of larval movement. Increased attention to local scales of coral genetic structure, dispersal, and selection may help increase the accuracy of models of future adaptation of corals and of assisted migration as a reef resilience intervention.

## 1 INTRODUCTION

Restoration of ecosystems in decline due to climate change is increasingly incorporating strategies that promote resilience of local species and populations. These renewal strategies are based on data suggesting which species and populations are likely to thrive in local regions over the next century, and may include non‐native taxa (Harrison, 2021) or genes for different traits (Borrell et al., 2020; Browne et al., 2019; Selmoni et al., 2020). For some forestry or seagrass communities, it may be possible to replace entire populations (Tan et al., 2020). However, in many other systems increased climate resilience may be best achieved through assisted gene flow – adding resilient individuals that increase the local frequency of alleles for resilient traits (Isabel et al., 2020; Van Oppen et al., 2015).

These more resilient individuals could help stabilize local populations, but their main value is likely to be in the production of local offspring that inherit genes for climate resilience, thereby increasing stress resilience in future generations (e.g., Aitken & Whitlock, 2013). Such positive benefits can be tempered by tradeoffs such as outbreeding depression, especially if imported individuals are from highly divergent populations, or cryptic species with intrinsic genetic incompatibilities (Frankham et al., 2011). Benefits also depend on the spatial scale of offspring dispersal and the scale of environmental heterogeneity. Low dispersal can allow resilience genes to locally accumulate, but can limit them from spreading between populations.

Reef building corals have been a focus of increasing restoration efforts because they are affected so profoundly by ocean warming stemming from climate change (e.g., Hughes et al., 2018), in addition to many other anthropogenic disturbances (e.g., Richmond et al., 2018). To address these mounting concerns, a suite of approaches has been suggested for interventions to increase climate resilience in corals (reviewed in National Academies of Sciences, Engineering, and Medicine, 2018), including using assisted gene flow to support local populations by adding climate adaptive alleles (Cunning et al., 2021; Drury & Lirman, 2021; Matz et al., 2020; Van Oppen et al., 2015) and protecting reefs with particular thermal and population traits to further population adaptation (DeFilippo et al., 2022; Palumbi et al., 2014).

Recent models of coral‐assisted gene flow in concert with different levels of conservation using marine protected areas suggest the potential for increased fitness in the face of moderate climate change (Bay et al., 2017; McManus et al., 2020; Walsworth et al., 2019). These models consider dispersal in many different ways. For example, Quigley et al. (2019) use oceanographic simulations to estimate larval movement in their models (see also Hock et al., 2017; Treml et al., 2008, 2012). Others (e.g., McManus et al., 2020; Walsworth et al., 2019) assume significant retention of coral larvae on natal reefs as well as stepping‐stone movement to adjacent reefs. These patterns of larval retention and local gene flow help establish the spatial scale over which assisted gene flow or reef protection will be an effective management strategy.

The need for dispersal estimates in predictions of future adaptation suggests a focus on measurement of small‐scale coral dispersal. To date, data from coral population genetics show a wide variety of scales of differentiation across species, locations, genetic markers, and environmental conditions (e.g., Ayre & Hughes, 2000; Shanks et al., 2003; Sheets et al., 2018). These data sets tend to focus on coral populations separated by 100s–1000s of km (Sheets et al., 2018) and assume that genetic differences are at equilibrium established by the balance between gene flow and genetic drift (Waples, 1998), which may take hundreds or thousands of generations to achieve. Small‐scale genetic structure from reef to reef over small numbers of generations is much less commonly studied in broadcast spawning corals (e.g., Gorospe & Karl, 2013; van der Ven et al., 2021). In contrast, close‐kin genetics and otolith chemistry have shown low effective dispersal for reef fish larvae on short time scales (e.g., Fobert et al., 2019; Jones et al., 1999), which has dramatically reshaped our perception of how far larval fish typically disperse over the course of a single generation.

Here, we take advantage of low‐pass genome sequencing of 284 corals of the species *Acropora hyacinthus* in Palau and use the short reads from this data set to re‐assemble complete mitochondrial genomes in order to test for patterns of local larval dispersal in this species. Despite low pass sequencing, the high multiplicity of mtDNA in animal cells allows us to assemble whole mitochondrial genomes with high coverage. In particular, the complete linkage of all bases in the mitochondrial genome allows this genome to be analyzed as a single highly polymorphic locus exhibiting a mutation rate thousands of times higher (when integrated across all 18,642 kb of the mitochondrial genome) than for a single SNP. The linkage of the bases in the mitochondrial genome allows us to test if any differences are primarily seen in the geography of divergent phylogenetic clades that have been in the population over evolutionary time scales, or among highly similar haplotypes with recent divergence times.

Our data show that very closely related mitochondrial genomes, no more than one base different, occur together on the same Palauan reefs more often than expected by chance, suggesting that low levels of dispersal result in haplotypes which are locally more abundant on single reefs. At the same time, comparison to data from American Samoa not only shows strong population differences but also revealed identical genomes in both locations in three cases. Both patterns together not only are consistent with local retention and selection of some coral larvae on natal reefs over short spatial scales but also the occurrence of rare long distance larval dispersal across the Pacific basin. These results help determine the scales of effective dispersal that are fundamental to models of future adaptation of reefs during climate change, and may help indicate whether local management decisions such as assisted migration might be able to increase adaptive potential (Colton et al., 2022; Matz et al., 2020).

## 2 METHODS

### 2.1 Sampling on reefs and in regions

These data originate from corals collected for testing regional patterns of bleaching resilience as described in Cornwell et al. (2021). Briefly, up to 10 colonies of the species *Acropora hyacinthus* were collected from each of 29 patch reefs within the lagoons of Palau, as well as from 10 locations on western fore reefs. Patch reefs were sampled from Northern and Southern lagoons which experience different patterns of oceanic currents (Golbuu et al., 2012) as well as a set of Western patch reefs in a broad shallow part of the lagoon, and Eastern patch reefs where waves and weather from the east are the dominant forces shaping the habitat. These regions were broadly defined by patterns of currents, wave exposure, depth, and temperature profile, although within‐region variation remains strong in some cases. In one departure from the regions in Cornwell et al. (2021), here we have separated the northern lagoon patch reefs into two regions: with the most northern patch reefs (Reefs 40–44) denoted NN and the rest (30–37) called N. In addition, we sampled 10 fore reef regions along the western edge of the barrier reef. Subsequent heat resistance and temperature profiling (Cornwell et al., 2021) showed that fore reefs on the western edge of the Northern lagoon (near Ebiil Channel) and those on the western edge of the Southern lagoon (near Ulong Channel) showed different environmental and heat resistance profiles, and so we analyzed these fore reef locations as different regions. Overall, reefs within regions are separated by 1–10 km, with the exception of the Ebiil fore reef locations, some of which are 11–14 km apart (Figure S1).

### 2.2 DNA isolation and sequencing

DNA was extracted from coral branches preserved in RNALater using a modified CTAB extraction protocol. Briefly, tissue was airbrushed off of the coral skeleton and pelleted in a 1.5 ml tube. After removing the supernatant, 600 μl of CTAB + 1.2 μl Beta‐mercaptoethanol + 2 μl Proteinase K were added to each sample which were digested at 55°C for 15 min. 600 μl of Chloroform:Isoamyl Alcohol (24:1) was added to each sample, mixed for 1 min and spun at max speed for 10 min on a table top centrifuge, after which the aqueous layer was transferred to another tube. This was repeated for a total of two times, after which point the DNA was precipitated using 600 μl of chilled isopropyl alcohol followed by two 70% ethanol washes (with centrifugation steps to pellet the sample after step). DNA quality was assessed using gel electrophoresis, degraded samples were cleaned using AmpureXP beads (0.7×). Purified DNA was then used to make indexed libraries which were run on the Illumina 4000 and 6000 platforms.

### 2.3 Mapping to mitochondrial genomes

Alignments to the *Acropora millepora* mitochondrial genome were performed with HISAT2 using default settings (Fuller et al., 2020; Kim et al., 2019). Coverage of the mitochondrial genome was calculated with Samtools depth, only including reads with an alignment quality score ≥ 30 (Li et al., 2009) and Phred score > 30 (Bokulich et al., 2013). Genotypes for every position on the genome were called using Bcftools multi‐allelic caller with ploidy set to one (Li, 2011). Only reads with an alignment quality ≥30 were utilized for genotype calling. Full mitochondrial genome sequences for each colony were assembled based on the genotype calls at each locus. Loci that did not have a called genotype are represented by “N” in a given assembly. In addition, we mapped these short reads to the *A. millepora* genome (ca. 0.4–0.6 X coverage) and compared high coverage SNPs to check for clonal identity. All of our corals are genetically distinct: none derive from the same gene.

Sequencing was done in three blocks after three separate rounds of DNA isolation on three different dates by the same outside sequencing vendor. The high read depth (average > 500) leaves little room for sequencing lane artifacts – one of the advantages of this approach. The best evidence for lack of lane effects is that corals with identical haplotypes were sequenced in different lanes – all haplogroups included corals from at least two lanes, usually all three.

### 2.4 Sequence comparison and haplogroups

Full mitochondrial sequences were aligned to one another and to 70 full mitochondrial sequences from other Pacific *Acropora* species downloaded from GenBank using MAFFT (Katoh et al., 2019). The aligned fasta file was imported into MEGAX and the pairwise distance matrix created.

All sequences showing 100% similarity were noted and assigned a Haplotype Group (1–31). There were 248 sequences in these 31 Haplogroups, as well as 31 sequences that differed from a particular Haplogroup by a single base that we folded into their respective Haplogroups.

We gathered representatives of all Haplogroups, along with the downloaded full mitochondrial genomes from congeners and generated a phylogenetic tree with Mega X (Maximum Likelihood with 100 bootstrap replicates, Kumar et al., 2018). We analyzed the geography of mitochondrial genomes by measuring PhiST values across reefs and regions (clusters of reefs within 1–14 km of one another in a particular part of the archipelago, see Cornwell et al. (2021) using GenAlEx6.4. PhiST is an analog of FST and is used as an index of genetic differentiation for DNA sequence haplotypes (Excoffier et al., 1992). We analyzed patterns of small‐scale genetic identity by scoring the reef and region for all individuals in each Haplogroup, and counted the number of instances in which individuals on the same reef shared an identity group.

We evaluated the significance of this small‐scale structure by permuting individuals in our data set randomly among reefs, and estimating the probability that the number of cases of shared Haplogroups on the same reef that we observed could have been generated by chance. We estimated *p*‐values by comparing the observed number of shared Haplogroups with the number observed in 1000 randomized simulations. These simulations compared our data with fully randomized colonies.

## 3 RESULTS

### 3.1 Low levels of genetic variation

We mapped short read sequences from whole genome libraries to the *Acropora millepora* mitochondrial genome in order to document the geographic distribution of whole mitochondrial genome sequences from 284 colonies of *A. hyacinthus* from Palau. On average our mapped mitochondrial genomes contained only 49 non‐determined bases out of over 18,000. Average read depth for the mitochondrial genomes was 535x (median = 332; standard deviation = 634). Despite this high coverage and large sample sizes, the number of variable nucleotide positions is low. We found 113 variable nucleotide positions among 18,482 bases in the mitochondrial genome, and on average individuals differed only at nine bases (~0.05%).

Within our *A. hyacinthus* samples, the most divergent sequences differed by 27–31 bases from the bulk of our samples. However, dominating our data sets are clusters of identical and highly related sequences, including 248 corals that fit into 31 Haplogroups. All of these 248 corals within their Haplogroups share identical mitochondrial genomes. Another 31 corals are a single unique base divergent from one of these Haplogroups, and are included within them for our analysis because these genomes are uniquely and highly related to the other sequences in the Haplogroup. Only five mitochondrial genomes neither fit into a Haplogroup nor are a single base divergent: these are on average 4.6–5.4 bases different from the Haplogroups.

**FIGURE 1:**
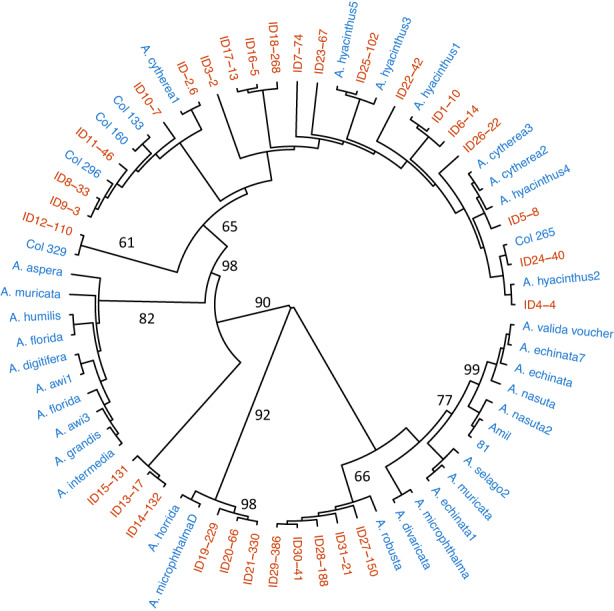
Phylogeny of full mitochondrial genomes of *A. hyacinthus* from Palau (red labels) compared to full mitochondrial genomes from other Pacific *Acropora* species gathered from GenBank. Percent bootstrap values from maximum likelihood trees are shown.

The phylogenetic tree of all 284 sequences (see map, Figure S2) plus whole mitochondrial genome sequences from other *Acropora* species show that most of the Palau sequences cluster closely with other *A. hyacinthus* and *A. cytherea* mitochondrial genomes (Figure 1). These sequences are in a clade with moderate bootstrap support (65%, Figure 1) and are related to a clade of sequences from nine other *Acropora* species. In addition there is a distinct clade (labeled ID27, 28, 29, 30, 31 in Figure 1) that is a sister clade to nine additional species including *A. millepora*, *A. selago, A. echinata*, and *A. valida*, and a second divergent clade (ID19, 20, 21 in Figure 1) clustering with sequences from *A. horrida* and *A. micropthalma*. Most *Acropora* species in current databases are represented by only a handful of complete genomes, and it is possible that a more complete dataset of genetic variation within each species would reveal more shared clades between species.

### 3.2 Geographic structure

We characterized the location of each coral by reef and by region, as in Cornwell et al. (2021). We used Phi_ST_ values (from GenalX6.4) to test whether the genetic variation within and between reefs, and within and between regions, was higher than expected by chance. The geographic component of mtDNA variation at the regional level was 2.0% (Table 1, PhiRT = 0.019, *p* = 0.02) primarily due to higher levels of genetic diversity resulting from the more divergent genotypes in the data set (Haplogroups ID27–31): these are more common at far Northern and fore reef locations and absent at all but two Southern, Eastern and Western reefs (Figure S2), although it is possible that a larger sampling effort would show that they are also present in eastern reef locations. Variation at the reef level was non‐significant (Table 1; PhiPR = −0.007, *p* = 0.66).

**TABLE 1:** Phi_ST_ AMOVA table comparing distribution of genetic diversity among mitochondrial genomes at the regional and reef levels

Source	Df	SS	MS	Est. var.	%
Among Regions	6	29.365	4.894	0.055	2%
Among Reefs	31	84.629	2.730	0.000	0%
Within Pops	246	707.795	2.877	2.877	98%
Total	283	821.789		2.933	100%

Stat	Value	*p* (rand > = data)
PhiRT (among regions)	0.019	0.020
PhiPR (among reefs)	−0.007	0.660
PhiPT	0.012	0.230

Abbreviations: %, Percent variance explained at this level; df, Degrees of freedom; MS, Mean squares; SS, Sum of squares.

### 3.3 Mitochondrial genome Haplogroup distribution

Compared to Phi_ST_, a different signal of small‐scale population structure can be estimated from the geography of highly similar mtDNA sequences. We found multiple colonies from the same Haplogroup on the same reefs 59 times, with as many as four colonies from a particular haplotype co‐localized on a particular reef (Figure S3). We expect common Haplogroups to occur more than once on some reefs; for example, our most abundant Haplogroup (Group 4 with 55 colonies) shows more than one colony on 18 reefs. Haplogroups comprised small numbers of individuals should be co‐located much more rarely, yet these are often found together. For instance, for both Haplogroups 15 and 31, the two colonies in each group are unlikely to be found together by chance alone. However, in both cases, the two colonies with the same mitochondrial genome are found on the same reef. Overall, of the 24 Haplogroups with small numbers of colonies (*N* = 2–10), 11 of the groups have colonies on the same reef (Figure S3).

**FIGURE 2:**
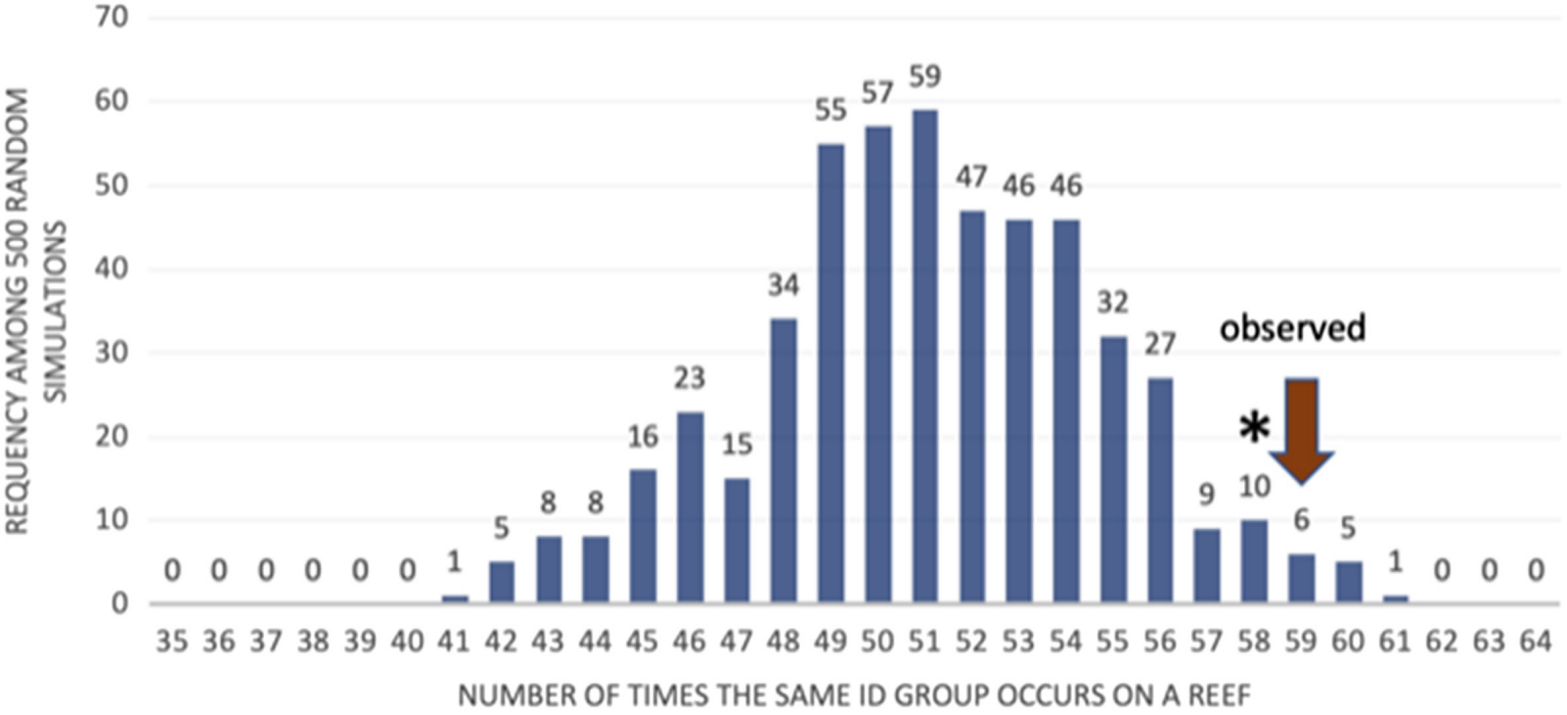
The number of times we see Haplogroups on the same reefs in simulations of random dispersal among reefs (of 1000 simulations). The red arrow represents the observed number of co‐occurrences from our data.

To estimate how many instances of co‐occurrence within Haplogroups we should expect by chance alone, we randomized reef location among corals, keeping the same number of Haplogroups, the same distribution of group sizes and the same number of colonies per reef. On average, randomization of the colonies resulted in 51 instances where corals within identity group were on the same reef (Figure 2, SD 3.7): the chance of seeing 59 pairs after randomization was *p* = 0.024. A similar pattern was seen across the subset of Haplogroups that are rare in the data set (with 2–10 colonies): randomizations show pairs of these colonies on the same reef an average of 3.08 times instead of the 11 we observe (*p* = 10^−3^).

We see parallel patterns when we exclude singletons from the analysis and compare strictly identical genomes, though lower sample sizes reduce the significance. The observed occurrence of identical haplotypes on the same reef across all haplotypes was 49 versus 43 expected (one‐tailed *p* value = 0.041). The observed co‐occurrence for rare genotypes (*N* = 2–10) was 7 versus 2 expected (*p* value = 0.002).

Despite the significant co‐occurrence of Haplogroups on reefs, Haplotypes had no significant regional clustering. Because observed and expected occurrences of most Haplogroups were higher for reefs grouped into regions, we could apply standard chi‐square tests: the distribution of 30 of 31 Haplogroups were not significant after multiple test corrections. (Chi Square Test, *p* = 0.36, see Table S1). Haplogroup 27 showed a chi‐square *p* value of 10^−4^ but was one of the groups with low occurrence (*N* = 3).

### 3.4 Isolation by distance effects

The reefs we studied are largely patch reefs less than a kilometer across (see associated video). In addition, reefs within regions are within 1–14 km of one another. As a consequence, these results suggest that our signal of genetic similarity decays quickly over very small scales. To test this, we regressed average nucleotide distance and average fraction of corals in Haplogroups between reefs against the geographic distance between reefs (Figure S4). There was a slight positive relationship between nucleotide distance and geographic distance, and a slight negative relationship with haplotype identity, but neither were significant. The strongest pattern was seen for Haplogroups showing small numbers of individuals (10 or less). This is similar to the increased significance of randomization tests for this subset of Haplogroups (see above).

### 3.5 Temperature effects

We also performed multiple linear regressions of nucleotide distance versus geographic distance and difference in temperature among reefs (measured as the fraction of time intervals above 31°C as in Cornwell et al., 2021). Again, we found that there was a slightly significant relationship among these variables (*p* = 0.046) but that the adjusted R^2^ was very low and explained very little of the variance (R^2^ = 0.006, Table S2). Together, these analyses suggest that the signal of genetic similarity we see is a function of co‐location on the same reef.

### 3.6 Relationship to ocean modeling

Golbuu et al. (2012) developed current flow models for Palau and used them to predict which areas of the archipelago have high coral recruitment and which have high self‐seeding. We used their maps to record recruitment and self‐seeding rates for the 39 reefs that we have surveyed here. Comparing these estimates of coral dispersal to the fraction of corals that are co‐located with other corals in the same Haplogroup on the same reef shows that (1) most of our reefs (32 of 39) are in areas predicted to have very low self‐recruitment, and (2) there are no significant relationships of these Haplogroup values to predicted rates (Figure S5).

### 3.7 Long distance patterns: Palau versus American Samoa

Recently, Rose et al. (2021) generated low pass, whole genome data from 155 colonies of *A. hyacinthus* from Ofu Island in American Samoa (NCBI Sequence Read Archive accession PRJNA657822). We mapped these reads to the mitochondrial genome of *A. millepora* and compared the assembled full mitochondrial genome sequences to our sequences from Palau. The data allow us to test the occurrence of clades and Haplogroups across a broad expanse of the Pacific and across a significant part of the species range of *A. hyacinthus*.

**FIGURE 3:**
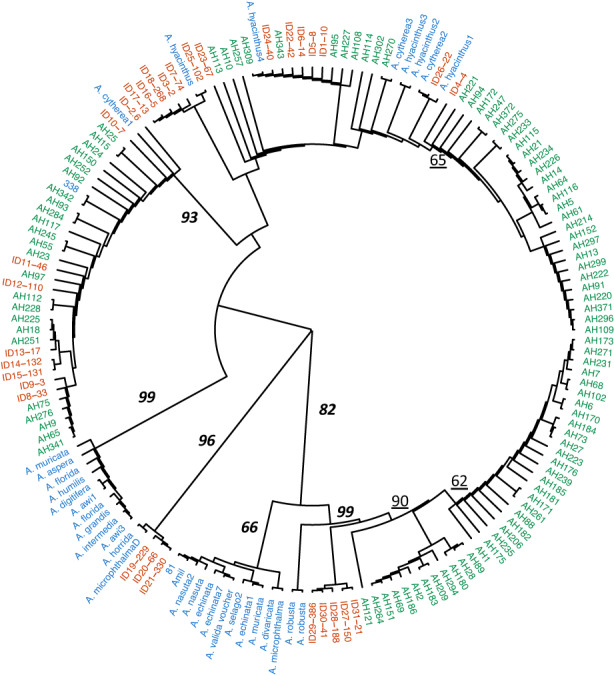
Mitochondrial Haplogroups from Palau (labeled in red with ID number as in Figure 1) and American Samoa (labeled AH in green), along with seven Palau sequences that are not in Haplogroups (blue labeled COL) and congeneric species (also blue). Major branch bootstrap values from maximum likelihood trees are shown when they are above 60%: Underlined values denote major monophyletic clades within species; bold‐italic branches denote polyphyletic clades.

Broadly, the American Samoa data fall into three major clades (green labels in Figure 3) that also include our Palau corals. These three clades include American Samoa sequences plus (1) the major outgroup clade from Palau identified in Figure 1 (ID27–31, Figure 3), (2) 14 different Palau Haplogroups interspersed with *A. hyacinthus* and *A. cytherea* sequences from GenBank, and (3) Palau ID8–15 (Figure 3). There is also one major clade of American Samoa sequences that does not include sequences from any other location. Despite the broad phylogenetic overlap, Phi_ST_ values are strongly positive across this distance (Phi_ST_ = 0.257, *p* < 10^−7^). In previous data sets, nuclear polymorphisms also showed strong differences from Palau to Samoa (*F*_ST_ = 0.059), as expected for comparison of coral populations more than 1000 km apart (Sheets et al., 2018).

In addition, some colonies from American Samoa show identical sequences to those of large Palau Haplogroups: eight colonies from American Samoa had sequences identical to Haplogroup ID10 (AH 15, 24, 25, 92, 117, 150, 252, 342), four had sequences identical to Haplogroup ID8 (AH 9, 65, 341, 276), and eight had sequences identical to Haplogroup ID26 (AH 95, 113, 114, 227,257, 270, 302, 309). All other large Haplogroups in Palau were exclusive to Palau locations.

## 4 DISCUSSION

Complete mitochondrial genome data from low coverage genome sequencing provide evidence of small‐scale population differentiation from reef to reef in Palau table top corals. Such genetic structure is visible both in the differential distribution of divergent lineages and in the occurrence of highly similar sequences on the same reef more often than expected by chance. The higher resolution of full length mitochondrial sequences, where identical sequences represent alleles inherited from a recent common ancestor, allows finer resolution of population patterns. Such small‐scale patterns could be generated by low larval movement and also by selection for larvae produced by local parents. These processes have been incorporated into recent models of coral adaptation in the face of climate change (e.g., DeFilippo et al., 2022; McManus et al., 2020) but such data are not often collected in traditional analyses of coral population genetics (Sheets et al., 2018).

### 4.1 Mitochondrial coverage and nucleotide diversity from whole genome sequencing

Mitochondrial genomes occur in 100s or 1000s of copies per cell (Wilson et al., 1985), and can make up as much as 0.5%–5% of the DNA content for corals with 400 Mb genomes. Mapping short genome reads onto known mitochondrial sequences results in average coverage of 535×, with high variance. This high coverage allows us to identify Haplogroups with identical genome sequences, including a small set of sequences with single, unique base pair differences, allowing us to map highly related maternal lineages across reefs and regions.

Anthozoans, including corals and anemones, are particularly known for low genetic diversity of mtDNA (Chen et al., 2009; Kitahara et al., 2010), especially in conserved genes such as COI. Similarly, our full mitochondrial genome sequences also have low amounts of sequence variation. On average our sequences differed by only 9.2 bases (0.05%) within populations of *A. hyacinthus*, and there are polymorphisms at only 113 of 18,642 bases. Variation across species is higher (Figure 1) but even when we include outgroup mitochondrial genomes from 20 other *Acropora* species, there are variable bases at only 211 nucleotide positions.

### 4.2 Mt phylogenies between and within species

The data allow us to phylogenetically compare whole mitochondrial genomes within and across species. Our sequences from Palau and American Samoa span nearly the entire phylogenetic range shown by the congeneric *Acropora* in our data set (Figure 1). For example, two clades of *A. hyacinthus* sequences are each more closely related to other species than to other conspecifics (Figure 3). In addition, two well defined clades include mitochondrial genomes from several other species (Figure 3). However, the sampling of these species is low, and further research might show that some of these species have multiple mitochondrial genome sequences scattered across the genus phylogeny.

Polyphyletic species groups based on mtDNA have been widely reported in Scleractinian corals at family (Kitahara et al., 2010), genus (Van Oppen et al., 2001), and species levels (Kitchen et al., 2021; Ramírez‐Portilla et al., 2021). In particular, complex polyphyly and monophyly have been seen in many other studies of the genus *Acropora* (see Van Oppen et al., 2001). In some cases, this might be due to cryptic species being lumped together in phylogenetic analyses. However, in other cases, polyphyly is likely due to introgressive hybridization among species that synchronously spawn and whose large effective populations sizes slow rates of genetic drift (Van Oppen et al., 2001).

Within species, Palau sequences are interspersed with American Samoa sequences, and with *A. hyacinthus* and *A. cytherea* sequences from other studies (Figure 3). However, there are also clades that hold sequences from one location but not both. For example, a large set of American Samoa sequences (from about 1 o'clock to 5 o'clock on the circular Figure 3) occur in one large clade found only in our American Samoan samples. Likewise, Palau Haplogroups ID19–21 and ID27–31 are in clades that have only Palau representatives.

### 4.3 Long distance comparisons

The major clades of mitochondrial genomes found in Palau are also seen in *A. hyacinthus* from American Samoa, reflecting their long phylogenetic history and wide dispersal. These clades are about 0.2% different, suggesting a diversification about 2 million years ago at an average rate of 0.1% per million years (Chen et al., 2009; Romano & Palumbi, 1997). As a consequence, it is not a surprise to see these ancient groups distributed across the Pacific.

More surprising is the occurrence of identical mitochondrial genome sequences from three separate Haplogroups in Palau and American Samoa. Because the average wait time for a mitochondrial genome mutation to occur is approximately 53,000 years (at 0.1% per million years), significant gene flow across the Pacific over this time frame is needed in order to account for the occurrence of these Haplogroups in both locations. This is relatively rapid colonization for population genetic processes, but is a long time by management standards. In addition, most Haplogroups are not represented in both locations, even the largest ones, suggesting that dispersal over this 53,000 year time frame is rare for mitochondrial lineages. Such information is not deeply surprising for locations separated as far apart as Palau and American Samoa (6400 km). However, the tools we develop here might be deployable to answer similar questions about long distance gene flow between other localities that are closer and that might be considered as candidates to seed other reefs (Quigley et al., 2019).

### 4.4 Haplogroup geography and small‐scale gene flow

Among Palau corals, two signals of population genetic distinction were apparent. In the first, reef regions separated by 40–55 km differed in the proportion of individuals with divergent mitochondrial genomes, showing a Phi_ST_ value of 0.02 (*p* = 0.02). These values are driven by the occurrence of a rare clade with strong divergence, and so the values of Phi_ST_ are driven by significant differences in frequency of this clade (Figure S2).

The second signal of population differentiation was the high number of colonies with the same Haplogroup on the same reef. This occurred 59 times in our data set, whereas a randomization test showed that without any geographic structure we would see such co‐location 51 times (SD 3.7). This pattern was particularly strong for smaller Haplogroups.

In Palau, current patterns during the predicted larval phase suggest that movement among reefs on the scale of our genetic sampling should be common. For example, many larvae produced in the northern lagoon of Palau were modeled as likely moving offshore to the west of the archipelago (Golbuu et al., 2012). Furthermore, most of the reefs we sampled are in locations that prior models suggest have low larval retention potential (figure 6 in Golbuu et al., 2012) and so our data on higher than expected co‐location rates at these reefs is surprising. For reef fish, close kin parentage studies have revealed surprisingly short dispersal distances in some cases. These studies also help parameterize biophysical models of dispersal and have shown better agreement when larval behavior is taken into account (Bode et al., 2019). Similar comparisons have not been done for corals, but could help resolve the differences we see in Haplogroup abundance and model results.

Two facets of our study may have made finding such small‐scale patterns more likely. First, we compare populations on many nearby reefs, whereas most coral genetic studies have not examined population genetic patterns across spatial scales as short as 1–14 km. Second, we are targeting recently evolved alleles, including identical mitochondrial genomes. Because bases in the mitochondrial DNA are fully linked to one another, comparing these sequences is essentially comparing a single genetic locus with 18,642 times the rate of mutation of a single base pair. Population genetic analyses of alleles with high mutation rates can provide a view of gene flow that is more contemporary than for low mutation alleles – this is particularly a problem in corals which have low mutation rates per base (Van Oppen et al., 2001). Especially when a mitochondrial Haplogroup is rare in a population, higher than expected local abundance may signal recent local retention of maternal lineages.

### 4.5 Scales of dispersal, selection, and genetic differentiation in corals

Coral genetic structure has often been shown over small spatial scales for brooding species that have short‐dispersal larvae but much less so for species like *Acropora* that broadcast gametes (Ayre & Hughes, 2000; Oliver et al., 1992; van der Ven et al., 2021). For broadcast species, significant differences tend to be over larger spatial scales. For example, SNP analysis of staghorn corals (*A. cervicornis*) along the Florida reef track shows significant differentiation only above 50–100 km (Drury et al., 2017). Similar geographic scales of genetic differentiation have been seen in massive *Montastrea* corals in Florida (Drury et al., 2020). Other cases of coral differentiation across 100s of km are common (e.g., Devlin‐durante & Baums, 2017; Matz et al., 2020; Nishikawa, 2008; Selmoni et al., 2020).

Studies at the kilometer scale for such corals are much less common. van der Ven et al. (2021) found slight differentiation of microsatellite markers in *A. millepora* across along a 25–35 km on‐shore/offshore gradient in Indonesia. Cros et al. (2016) showed substantial genetic differentiation from eastern to western fore reef locations in Palau, also using microsatellite markers. Our data show that single reefs in Palau have substantially higher occurrence of mitochondrial Haplogroups than expected, supporting the hypothesis that local population structure at the reef scale in *A. hyacinthus* is due to low effective migration rates.

Genetic differentiation can also be seen for corals living across environmental gradients such as depth or water quality where selection might be acting (Eckert et al., 2019; Tisthammer et al., 2020). For instance, Selmoni et al. (2020) compared polymorphism levels in *A. digitifera* across nearly 8000 SNPs from two broad sampling areas in Okinawa and the Yaeyama Islands approximately 500 km away, and found 18 loci that seemed to have a high association with coral bleaching alerts and with latitude. Similarly, Cooke et al. (2020) showed strong genetic differentiation in *A. tenuis* between locations on the Great Barrier Reef, with indications of outlier loci under selection. Over a smaller spatial scale, Bay and Palumbi (2014) recorded 100s of genomic SNP differences for corals in two adjacent back reef pools in American Samoa that have substantially different heat stress regimes. A different type of selection has been implicated in the association of coral hosts with their internal algal symbionts, especially when the symbionts confer differential response to environmental stresses such as heat (Thornhill et al., 2017). These data show that both distance and environmental variation (presumably along with natural selection) play a strong role in coral genetic differentiation at moderate to long distances.

Both selection and restricted dispersal could be acting to produce the patterns we see among mitochondrial genomes. A clearly important variable across the 39 reefs in our survey is the temperature difference among these reefs (Cornwell et al., 2021). These differences correlate with changes in heat tolerance among corals on the, yet variation in temperature has low power in explaining the genetic patterns we see (Table S2). Other possibilities include slight differences in depth, water motion, water quality, or symbiont community occurring across a fine scale spatial mosaic (e.g., Cornwell & Hernández, 2021; Thomas et al., 2018). In such cases, local parents may have been selected to have high fitness in the local environment for a variety of reasons, and their larvae might also have higher fitness on their natal reef.

Short dispersal occurs in many brooding corals, but broadcast corals such as in *Acropora* have longer larval durations and less genetic structure (see van der Ven et al., 2021 for a recent example). Models of movement of broadcast coral larvae typically show dispersal ranges of 100s of km (Matz et al., 2020; Quigley et al., 2019; Treml et al., 2012), and similar modeling work in Palau shows similar patterns of high dispersal and low retention on native reefs (Golbuu et al., 2012).

In such cases, local adaptation of corals can still occur through a combination of dispersal and selection each generation (e.g., Hilbish & Koehn, 1985), but the scale of that adaptation is likely to be set by the specifics of larval dispersal and the degree of selection. As a result, higher occurrence of locally produced larvae, and high apparent mtDNA identity, as we find here, could be driven by some degree of local retention of planktonic larvae through water currents, or by increased survival of those larvae compared to immigrants, or both. Whole Genome Sequence data from many independent SNPs across the genome could shed much needed light on patterns of differentiation across many other genes that play different roles in the coral life cycle.

### 4.6 Applications to local coral management and assisted gene flow

Interventions to increase the resilience of coral reefs include a wide variety of tools, but few of them have been extensively tested in the field (National Academies of Sciences, Engineering, and Medicine, 2018). Of particular promise is assisted gene flow, the idea of finding existing heat resistant corals, transplanting them to local reefs and thereby increasing the abundance of heat‐resistant genotypes and genes (van Oppen et al., 2015).

Modeling studies illustrate the potential value of colonies with these genes in creating future populations with higher heat resistance (Bay et al., 2017; Matz et al., 2020; McManus et al., 2020; Quigley et al., 2019; Walsworth et al., 2019). Quigley et al. (2019) showed buildup of heat resistance alleles at 100 km scales followed by spread in subsequent 100s of generations. Matz et al. (2020) developed an Indo‐West Pacific model of adaptation based on the migration models of Treml et al. (2012) and showed the possibility of long‐term adaptation across ocean‐wide environmental gradients. At a more local level, McManus et al. (2020) and DeFilippo et al. (2022) used stepping stone models with about 20% recruitment back to the natal reef and showed that wider dispersal spreads locally adaptive alleles but slows potential local increases in adaptive traits such as heat resistance. These models suggest a critical importance of the spatial scales over which natural selection increases allele frequencies to generate successful response to climate (Matz et al., 2020), and the scales over which assisted gene flow to accelerate that response will be most valuable (McManus et al., 2020; Quigley et al., 2019).

Our data across local reefs in Palau suggest the possibility that local larval dispersal or selection at small geographic scales are responsible for a higher fraction of recruits than expected by traditional models. A combination of limited dispersal and varied natural selection across local scales of 10s of km might allow the mechanisms of adaptation studied over longer distance (e.g., the Indo‐West Pacific, Matz et al., 2020) to also be observed on local reef complexes. In such cases, the value of local programs of assisted migration and reef protection might be able to be modeled and incorporated into local planning (Colton et al., 2022).

## ACKNOWLEDGEMENTS

This work was done in collaboration with PICRC, Koror, Aimeliik, Ngarchelong, and Kayangel states and the national government of Palau. We are grateful for their permission to study Palau's reefs and their sharing of knowledge and help. We also thank Vernice Yuzi of Palau Community College for continued interaction and encouragement of students. The project was supported by funds from the Chan‐Zuckerberg BioHub and NSF OCE‐1736736, as well as MAC3 and Stanford donors to the Strong Corals Initiative.

## CONFLICT OF INTEREST

The authors declare no conflict of interests.

## Supporting information


Appendix S1

## Data Availability

Data for this analysis are available as aligned full mitochondrial genomes in fasta format as Appendix S1, and on genbank. Metadata from these files include colony name, reef of origin, latitude and longitude, and date of collection.
