# Supplementary material for: Small‐scale genetic structure of coral populations in Palau based on whole mitochondrial genomes: Implications for future coral resilience
Source: Evol Appl. 2023 Jan 5;16(2):518–29. doi: 10.1111/eva.13509 (PMC9923468; doi:10.1111/eva.13509)
Supplement: Supplementary file 1 — Appendix S1 [file EVA-16-518-s001.pdf]

Supplemental results

Supplemental figure 1: Distribution of distances of reefs within regions and between regions.

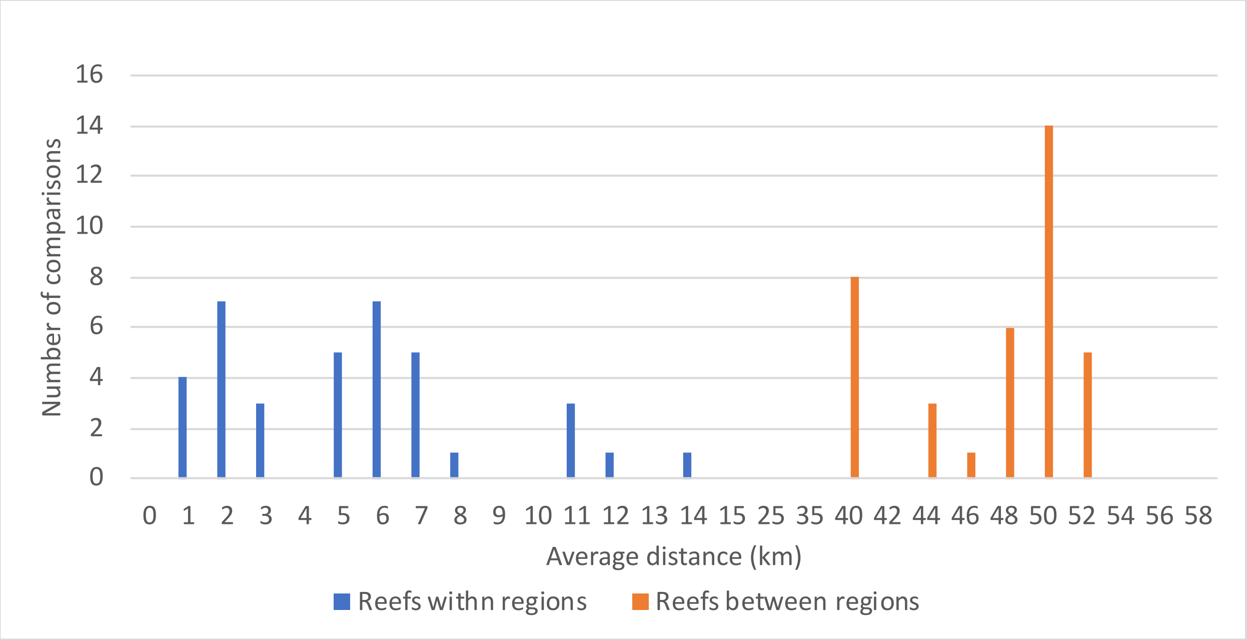

Supplemental Figure 2 : Geographic distribution of mitochondrial DNA diversity (sum of squares within populations) within reef regions in Palau. Upper: SSWR and sample sizes mapped to corresponding reef regions. Regions are denoted by colored boxes. Lower: same data shown comparatively across regions.

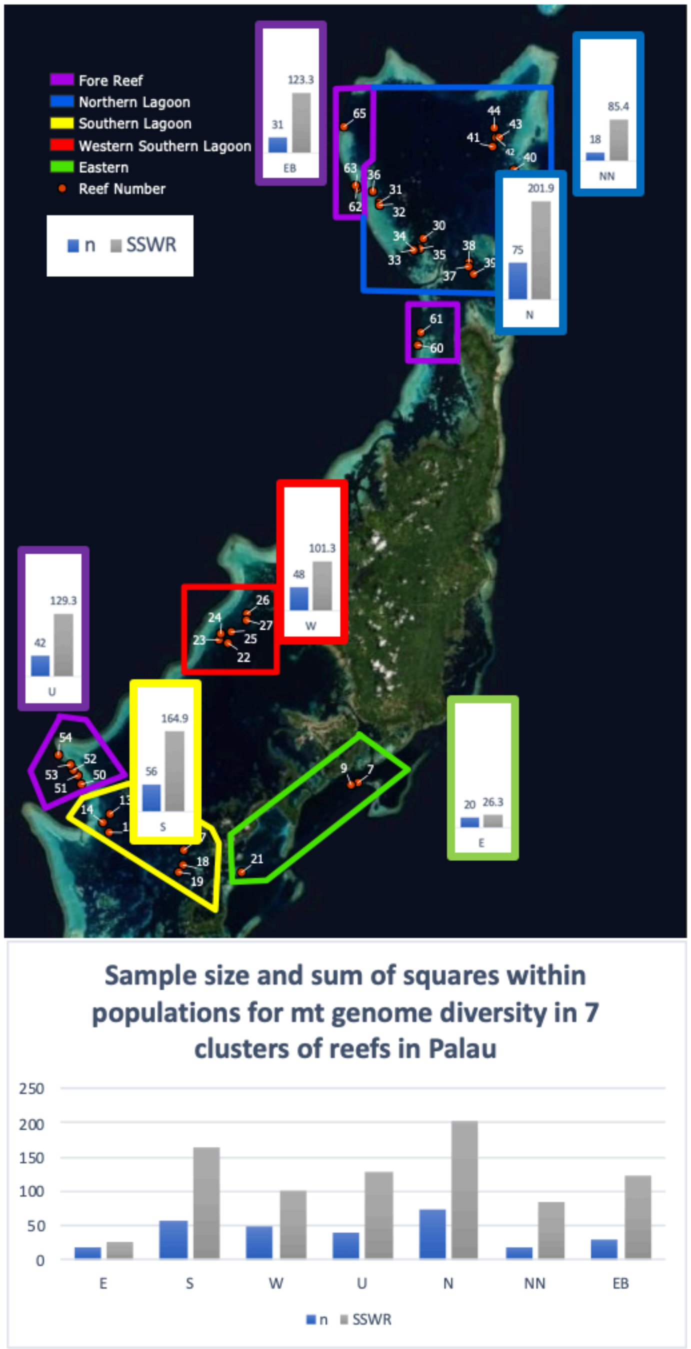

Supplemental Figure 3: occurrence of 31 mitochondrial genome Haplogroups across 40 reefs in Palau. Green squares denote no occurrence. Yellow squares denote one occurrence, and orange to red squares denote 2-4 occurrences. Reefs numbers are as shown in Suppl. Fig. 1 and Cornwell et al (2020). There are a total of 59 occurrences of more than one colony of an Haplogroup on an individual reef.

	Reef																																									# ID groups v
ID group	7	9	13	14	15	17	18	19	21	22	23	24	25	26	27	30	31	32	33	34	35	36	37	38	39	40	41	42	43	44	50	51	52	53	54	60	61	62	63	65	sum	59
1	1	0	0	0	0	0	0	0	0	0	1	1	0	1	1	0	0	1	1	0	0	0	1	0	1	0	0	0	0	0	0	0	0	0	0	0	1	1	0	0	11	0
2	1	0	0	0	1	0	0	0	0	0	0	2	0	1	0	0	0	0	0	0	0	0	0	0	0	0	0	0	0	0	1	1	0	1	0	0	0	0	0	1	9	1
3	1	2	0	1	2	1	0	0	2	1	0	0	2	1	3	3	1	1	0	2	0	0	0	1	0	0	0	0	0	0	0	0	2	0	0	2	2	1	0	1	32	10
4	2	3	3	2	2	1	3	2	2	0	1	3	1	0	0	3	0	1	2	1	2	1	2	0	3	2	1	1	0	0	2	2	1	1	1	2	1	0	0	1	55	18
5	1	0	0	0	0	0	0	0	0	0	0	1	0	0	0	0	0	0	0	0	0	0	0	0	0	0	0	0	0	0	0	0	0	0	0	0	0	0	0	0	2	0
6	0	2	0	0	0	0	0	0	0	0	0	0	0	1	0	0	0	0	0	1	0	0	1	0	0	0	0	0	0	0	0	1	0	0	0	0	0	0	0	0	6	1
7	0	0	0	1	0	0	0	0	0	0	0	0	0	0	0	0	1	0	0	0	0	0	0	0	0	0	0	0	0	0	0	0	2	0	0	0	1	0	0	1	6	1
8	0	0	0	0	0	1	0	0	0	1	2	0	1	0	0	0	0	0	0	1	0	1	0	1	0	0	2	0	0	0	1	0	0	1	0	0	0	0	0	1	13	2
9	1	0	0	0	0	0	0	0	0	0	0	0	0	1	0	0	0	0	1	0	0	0	0	0	0	0	0	0	0	0	0	0	0	0	0	0	0	1	0	0	4	0
10	1	1	1	0	1	0	0	1	0	1	0	0	0	0	0	0	2	1	1	0	0	1	1	0	0	0	0	0	1	0	1	0	1	0	2	0	0	0	0	0	17	2
11	0	0	0	0	0	0	1	0	0	1	0	0	1	0	0	0	1	0	1	0	0	1	0	0	0	0	0	0	0	0	1	0	0	0	1	0	0	1	0	1	10	0
12	0	0	0	0	0	0	0	0	0	0	1	0	0	0	1	0	0	0	0	0	0	0	0	0	0	0	0	0	0	0	0	0	0	0	0	0	0	2	0	0	4	1
13	0	1	0	0	0	0	0	0	0	1	0	1	0	0	0	0	0	0	0	0	0	0	0	0	0	1	0	0	0	0	0	0	2	0	0	0	0	0	0	0	6	1
14	0	0	0	0	0	0	0	0	0	1	0	0	0	0	0	0	0	1	0	0	0	0	0	0	0	0	0	0	0	0	0	0	0	0	0	0	0	0	0	0	2	0
15	0	0	0	0	0	0	0	0	0	2	0	0	0	0	0	0	0	0	0	0	0	0	0	0	0	0	0	0	0	0	0	0	0	0	0	0	0	0	0	0	2	1
16	1	0	0	1	0	1	2	3	1	0	0	0	1	0	0	2	1	2	1	0	4	2	1	2	1	1	1	0	0	0	0	0	1	0	2	1	1	0	0	1	34	8
17	0	1	0	0	0	1	0	0	0	0	0	0	0	0	0	0	0	0	0	0	0	0	0	0	1	0	0	0	0	0	0	0	0	0	0	0	0	0	0	0	3	0
18	0	0	0	0	0	0	0	0	0	0	0	0	0	0	0	0	0	0	0	0	0	0	0	2	0	0	0	0	0	0	0	0	0	0	0	0	0	0	0	0	2	1
19	0	0	0	0	0	0	0	0	0	0	0	0	0	0	0	0	0	0	0	1	0	0	0	0	0	0	0	0	0	0	0	0	0	0	0	0	0	0	0	0	1	0
20	0	0	0	0	1	0	0	0	0	0	0	0	0	0	0	0	0	0	0	0	0	0	1	0	0	0	0	0	0	0	0	0	0	0	0	0	0	0	0	0	2	0
21	0	0	0	0	0	0	0	0	0	0	0	0	0	0	0	0	0	0	0	0	0	1	0	0	0	0	0	0	0	0	0	0	0	0	0	0	0	0	0	1	2	0
22	0	0	0	1	0	0	0	0	0	0	0	0	0	0	1	0	0	0	0	0	0	0	0	0	0	0	0	0	0	0	1	0	0	0	0	0	0	0	0	0	3	0
23	0	0	0	1	1	0	1	0	0	0	0	0	0	0	0	0	0	0	0	0	0	0	0	0	0	0	0	0	0	0	0	0	0	0	0	0	0	0	0	0	3	0
24	0	0	0	0	0	1	0	0	0	0	0	0	0	0	0	0	0	0	0	0	0	0	0	0	0	0	0	0	0	0	0	0	0	1	0	0	0	0	0	0	2	0
25	0	0	0	0	0	0	0	0	0	0	1	0	0	0	0	0	0	0	0	0	0	0	0	0	0	0	0	0	0	0	0	0	0	0	1	0	0	0	0	0	2	0
26	0	0	3	1	0	0	2	0	3	0	1	2	1	1	2	0	0	0	0	1	2	0	0	0	0	2	1	1	0	0	1	2	0	3	0	2	0	1	0	0	32	10
27	0	0	0	0	0	0	0	0	0	0	0	0	0	1	0	0	0	0	0	0	0	0	0	0	0	2	0	1	0	0	0	0	0	0	0	0	0	0	0	0	4	1
28	0	0	0	0	0	0	0	0	0	0	0	0	0	0	0	0	0	1	0	0	0	0	0	0	1	0	0	0	0	0	0	0	0	1	0	1	0	0	0	0	4	0
29	0	0	0	0	0	0	0	0	0	0	0	0	0	0	0	0	0	0	0	0	0	0	0	0	0	0	0	0	0	0	0	0	0	0	0	1	1	0	0	0	2	0
30	0	0	0	0	0	0	0	0	0	0	0	0	0	0	0	0	0	0	0	0	0	0	0	0	0	0	0	0	0	0	1	0	1	0	0	0	0	0	0	0	2	0
31	0	0	0	0	0	0	0	0	2	0	0	0	0	0	0	0	0	0	0	0	0	0	0	0	0	0	0	0	0	0	0	0	0	0	0	0	0	0	0	0	2	1

Supplemental Figure 4: Average nucleotide distance (upper) and fraction of corals within identical Haplogroups (middle) as a function of the geographic distance (km). The fraction of corals within rare haplogroups (numbering 10 instances or less, lower panel) shows a stronger decline with distance, largely because of the drop from the zero distance category. The slight increase of nucleotide distance and declines of fraction identity across the geographic range are non-significant (p>0.5).

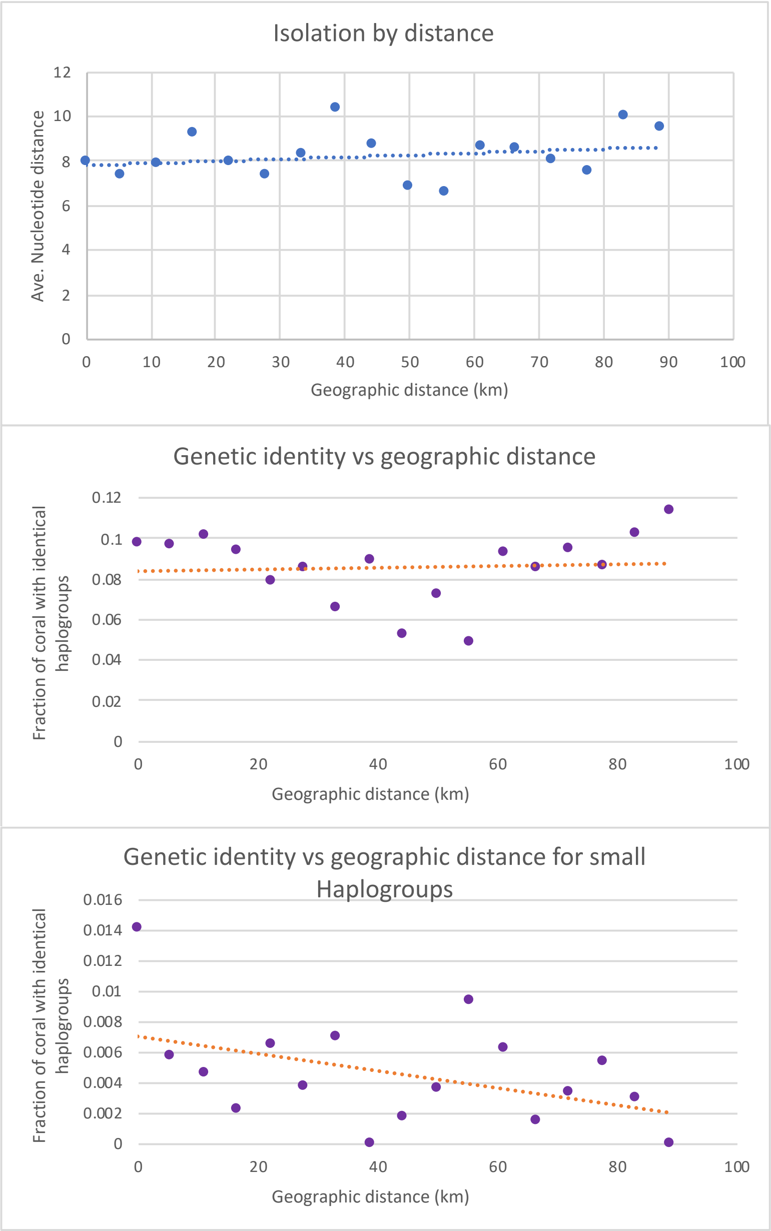

Supplemental Figure 5: Lack of relationship between the predicted degree of self-seeding (upper) and total coral seeding (middle) and coral cover (lower) at each of our 39 reefs based on current flow models in Golbuu et al. (2012) compared to the fraction of corals on those reefs that have another member of the same Haplogroup on the same reef. Most of the reefs in this study fall into zones where the published oceanographic models predict very little self-seeding, and so the power of these analyses to detect a relationship between self-seeding and haplotype identity rates is low.

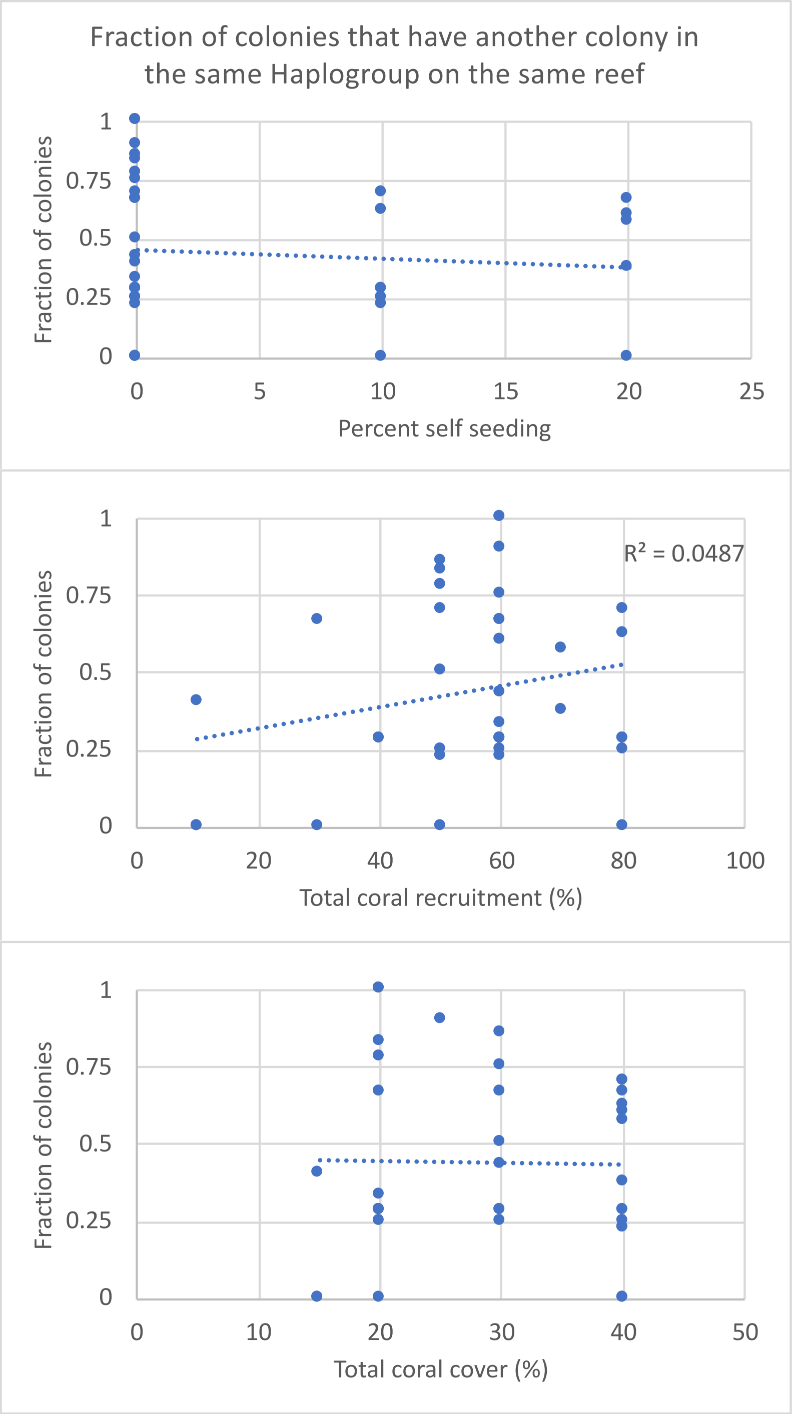

Supplemental Table 1: observed and expected occurrences of Haplogroups across regions:

ccurrences across regions								Expected occurrences								
Haplogroup	N	NN	S	E	W	U	Eb	sum	N	NN	S	E	W	U	Eb	chisq
1	4	0	0	1	4	0	2	11	2.84	0.53	2.17	0.84	1.95	1.46	1.20	0.23
2	0	0	1	1	3	2	0	7	1.81	0.34	1.38	0.54	1.24	0.93	0.76	0.27
3	6	0	6	3	7	1	5	28	7.23	1.35	5.53	2.15	4.97	3.73	3.05	0.44
4	14	3	14	5	4	7	4	51	13.16	2.47	10.08	3.91	9.05	6.79	5.55	0.44
5	0	0	0	1	1	0	0	2	0.52	0.10	0.40	0.15	0.35	0.27	0.22	0.21
6	2	0	0	2	1	1	0	6	1.55	0.29	1.19	0.46	1.06	0.80	0.65	0.23
7	1	0	1	0	0	1	1	4	1.03	0.19	0.79	0.31	0.71	0.53	0.44	0.89
8	3	2	1	0	3	2	1	12	3.10	0.58	2.37	0.92	2.13	1.60	1.31	0.34
9	1	0	0	1	1	0	1	4	1.03	0.19	0.79	0.31	0.71	0.53	0.44	0.67
10	5	1	3	2	1	4	0	16	4.13	0.77	3.16	1.23	2.84	2.13	1.74	0.61
11	3	0	1	0	2	2	1	9	2.32	0.44	1.78	0.69	1.60	1.20	0.98	0.81
12	0	0	0	0	2	0	2	4	1.03	0.19	0.79	0.31	0.71	0.53	0.44	0.39
13	0	0	0	1	2	1	0	4	1.03	0.19	0.79	0.31	0.71	0.53	0.44	0.27
14	1	0	0	0	1	0	0	2	0.52	0.10	0.40	0.15	0.35	0.27	0.22	0.77
15	0	0	0	0	2	0	0	2	0.52	0.10	0.40	0.15	0.35	0.27	0.22	0.11
16	14	1	8	1	0	2	3	29	7.48	1.40	5.73	2.22	5.15	3.86	3.16	0.02
17	1	0	1	1	0	0	0	3	0.77	0.15	0.59	0.23	0.53	0.40	0.33	0.55
18	2	0	0	0	0	0	0	2	0.52	0.10	0.40	0.15	0.35	0.27	0.22	0.35
19	0	0	0	0	0	0	0	0	0.00	0.00	0.00	0.00	0.00	0.00	0.00	#DIV/0!
20	1	0	1	0	0	0	0	2	0.52	0.10	0.40	0.15	0.35	0.27	0.22	0.81
21	1	0	0	0	0	0	1	2	0.52	0.10	0.40	0.15	0.35	0.27	0.22	0.89
22	0	0	0	0	1	1	0	2	0.52	0.10	0.40	0.15	0.35	0.27	0.22	0.50
23	0	0	2	0	0	0	0	2	0.52	0.10	0.40	0.15	0.35	0.27	0.22	0.16
24	0	0	1	0	0	0	0	1	0.26	0.05	0.20	0.08	0.18	0.13	0.11	0.56
25	0	0	0	0	1	1	0	2	0.52	0.10	0.40	0.15	0.35	0.27	0.22	0.50
26	3	3	8	0	7	5	3	29	7.48	1.40	5.73	2.22	5.15	3.86	3.16	0.12
27	0	2	0	0	1	0	0	3	0.77	0.15	0.59	0.23	0.53	0.40	0.33	0.00
28	2	0	0	0	0	1	1	4	1.03	0.19	0.79	0.31	0.71	0.53	0.44	0.65
29	0	0	0	0	0	0	2	2	0.52	0.10	0.40	0.15	0.35	0.27	0.22	0.88
30	0	0	0	0	0	2	0	2	0.52	0.10	0.40	0.15	0.35	0.27	0.22	0.03
31	0	0	1	0	0	0	0	1	0.26	0.05	0.20	0.08	0.18	0.13	0.11	0.56
	64	12	49	19	44	33	27	248	64.00	12.00	49.00	19.00	44.00	33.00	27.00	
27-31	2	2	1	0	1	3	3	12	3.10	0.58	2.37	0.92	2.13	1.60	1.31	0.19

Supplemental Table 2: results of Multiple Linear Regression of nucleotide distance against temperature and geographic distance.

```
Coefficients:
                               Estimate Std. Error t value Pr(>|t|)
(Intercept)                   7.620e+00  2.073e-01  36.761   <2e-16 ***
c(as.matrix(TemperatureDist)) 1.657e-01  8.768e-02   1.890   0.0592 .
c(as.matrix(GeographicDist))  4.573e-06  3.923e-06   1.166   0.2442
---
Signif. codes:  0 ‘***’ 0.001 ‘**’ 0.01 ‘*’ 0.05 ‘.’ 0.1 ‘ ’ 1

Residual standard error: 2.707 on 664 degrees of freedom
  (702 observations deleted due to missingness)
Multiple R-squared:  0.009191,    Adjusted R-squared:  0.006206
F-statistic:  3.08 on 2 and 664 DF,  p-value: 0.04663
```
